# Association of ANGPTL8 (Betatrophin) Gene Variants with Components of Metabolic Syndrome in Arab Adults

**DOI:** 10.1038/s41598-020-63850-7

**Published:** 2020-04-21

**Authors:** Amal Alenad, Mona M. Alenezi, Majed S. Alokail, Kaiser Wani, Abdul Khader Mohammed, Abdullah M. Alnaami, Maha Sulimani, Seema Zargar, Mario Clerici, Nasser M. Al-Daghri

**Affiliations:** 1 Biochemistry Department, College of Science, King Saud University, Riyadh, 11421 Saudi Arabia; 2 Chair for Biomarkers of Chronic Diseases, Biochemistry Department, College of Science, King Saud University, Riyadh, 11421 Saudi Arabia; 3 Sharjah Institute for Medical Research, University of Sharjah, 27272 Sharjah, United Arab Emirates; 4 Department of Physiopathology and Transplantation, University of Milan, via F.lli Cervi 93, Segrate, 20090 Milan, Italy; 5 IRCCS Fondazione Don Carlo Gnocchi ONLUS, Milan, 20148 Italy

**Keywords:** Peptide hormones, Metabolic syndrome

## Abstract

Angiopoietin-like protein 8 (ANGPTL8) has a role in lipid metabolism, beta-cell proliferation and diabetes progression, however, the association between different variants in the ANGPTL8 gene and metabolic syndrome (MetS) components has not been studied widely especially in Arab ethnic groups. In this study, the associations of ANGPTL8 variants on MetS risk in Saudi Arab adults were investigated. A total of 905 unrelated Saudi adults (580 healthy controls and 325 MetS) were included. MetS was screened based on the International Diabetes Federation (IDF) criteria. The genotype and allele frequency distribution of rs737337 (T/C) and rs2278426 (C/T) polymorphism in ANGPTL8 gene was studied. Participants with MetS were significantly older, had higher BMI, and rs737337 polymorphism frequency was significantly lower than in control. Furthermore, the TC + CC genotype and C allele of rs737337 (T/C) was associated with decreased risk of hypercholesterolemia and hyperglycemia [odds ratio (OR) 0.61, 95%CI 0.40–0.93, p = 0.016 and OR 0.58, 0.39–0.86, p = 0.007 respectively for hypercholesterolemia; and OR 0.66, 0.45–0.97, p = 0.032 and OR 0.65, 0.46–0.93; p = 0.016 respectively for hyperglycemia]. Similarly, CT, CT + TT genotype and T allele of rs2278426 (C/T) were associated with decreased risk of hyperglycemia (p < 0.05). In conclusion, the study suggests that the gene variants in SNPs rs 737337 (T/C) and rs 2278426 (C/T) are associated with lower risk of hypercholesterolemia and hyperglycemia. These findings supplement the growing literature supporting the role of ANGPTL8 in lipid and glucose metabolism.

## Introduction

Metabolic syndrome is a low-grade inflammation and oxidative stress state represented by a cluster of disorders including abdominal obesity, dyslipidemia, hypertension and insulin resistance^1^. This condition predisposes itself to vascular harm and subsequent manifestation of complications like cardiovascular diseases, type 2 diabetes mellitus (T2DM)^2^. MetS has a huge impact on the global health outcome, and the epidemic proportions of its prevalence^3^ demand research to reveal novel predictive factors showing association with MetS incidence and progression.

Angiopoietin-like protein 8 (ANGPTL8) or betatrophin, first identified in 2012, is a novel member of a group of secretory glycoproteins due to its role in lipid metabolism^4^. It is expressed abundantly in human liver, whereas in mice, its expression was shown to be high in liver, white and brown adipose tissues^5^. In humans, it is expressed predominantly in the liver^6^, suggesting that it might function as a hepatokine. Because of the controversy related to its function as a β-cell proliferator, many researchers in the recent past have tried to investigate the effects of betatrophin in beta-cell proliferation and diabetes expansion^7,8^. Nevertheless, while some suggest no role of betatrophin in beta-cell proliferation^9^, yet there are evidences in the literature which suggest that the factors related to metabolic diseases such as age, gender, waist circumference, total/HDL cholesterol ratio and renal function maybe associated with the circulating levels of betatrophin^10^ suggesting that it may play a role in the emergence and progression of metabolic diseases.

ANGPTL8 gene encodes for the secretory protein involved in the regulation of triglyceride (TG) levels through its interaction with betatrophin and inhibition of lipoprotein lipase (LPL) activity^11^. In betatrophin knockout mice studies, decreased levels of TG was reported^12^. Also, betatrophin overexpression has been associated with elevated circulating levels of TG^13^. According to Several GWAS studies, betatrophin/ANGPTL8 gene variants were associated with lipid phenotype and glucose homeostasis in humans^14,15^. There are three sequence variations of ANGPTL8 linked with lipid metabolism^16^: the SNP rs2278426 that represent a SNP (CGG to TGG) where amino acid arginine (R) is replaced by tryptophan (W)^15^. Minor allele frequency (MAF) of this SNP is T(W) and its percentage varies across ethnicities, it represents 15% in 1000 Genomic Project populations, 26% in Hispanics, 18% and 5% in African and European Americans respectively^14,15^. The second SNP rs737337 (T to C) results in a synonymous variant in DOCK6, upstream of the ANGPTL8 transcription start site^17^. The third SNP rs145464906 (CAG to TAG) results in a premature stop codon at residue 121^18^.

Previous studies have demonstrated associations between circulating levels of betatrophin and components of MetS; however such associations at the level of ANGPTL8 gene variants remain scarce especially in populations like Saudi Arabia where MetS prevalence is high. Taking into account the involvement of ANGPTL8 gene and its variants in lipid and glucose metabolism; the fact that alterations in lipid and glucose metabolism predisposes to metabolic disorders; and the scarcity of data on the associations of these gene variants on MetS and its components especially in Saudi population, we investigated the role of ANGPTL8 gene variants in MetS phenotypes among adult Saudis.

## Results

### General characteristics

The general characteristics of the study participants, divided into MetS group and control group, are presented in Table 1. As expected, participants with MetS were significantly older, had higher BMI, waist and hip circumferences, systolic and diastolic blood pressure, total cholesterol, triglycerides, fasting glucose, LDL-cholesterol and decreased HDL-cholesterol than those without MetS.

**Table 1:** Anthropometric and Metabolic Characteristics of subjects according to study groups.

Parameters	Control	MetS	P-Value
N (M/F)	580 (296/284)	325 (145/180)	
Age (Years)	40.2 ± 10.8	43.2 ± 9.9	<0.01
Body Mass Index (kg/m^2^)	26.7 ± 6.0	35.1 ± 4.2	<0.01
Waist circumference (cm)	90.2 ± 18.1	105.1 ± 15.5	<0.01
Hip circumference (cm)	99.9 ± 17.7	114.7 ± 15.9	<0.01
Waist-Hip Ratio	0.90 ± 0.12	0.91 ± 0.12	0.08
Systolic BP (mmHg)	116.1 ± 13.2	127.4 ± 13.7	<0.01
Diastolic BP (mmHg)	75.2 ± 9.1	80.6 ± 8.9	<0.01
Glucose (mmol/L)	5.4 ± 0.7	5.8 ± 0.7	<0.01
Triglycerides (mmol/L) #	1.3 (0.9–1.7)	1.8 (1.3–2.2)	<0.01
Total Cholesterol (mmol/L)	5.1 ± 1.1	5.4 ± 1.1	<0.01
HDL-Cholesterol (mmol/L)	0.9 ± 0.4	0.8 ± 0.3	<0.01
LDL-Cholesterol (mmol/L)	3.4 ± 0.9	3.7 ± 0.9	<0.01

Note**:** Independent sample t-test was used to compute the difference between studied groups. Data were presented as mean ± standard deviation. # indicates data presented as median.

### Genotype and Allele frequency distribution of the two SNP’s

The genotype and allele frequency distribution of SNP’s rs737337 (T/C) and rs2278426 (C/T) polymorphism in ANGPTL8 gene are presented in Table 2. The T-allele frequency of rs737337 polymorphism was significantly higher in controls than MetS participants (p = 0.02), however, the genotype/allele frequency distribution of rs2278426(C/T) was comparable in both the study groups (p > 0.05).

**Table 2:** Genotype and allele frequency distribution of rs737337 and rs2278426 SNPs for the individuals enrolled in the study.

Parameters	All	MetS	Control	P-Value
**rs737337 (T/C)**				
TT	756 (83.5)	280 (86.2)	476 (82.1)	0.10
TC	134 (14.8)	43 (13.2)	91 (15.7)	
CC	15 (1.7)	2 (0.6)	13 (2.2)	
TC + CC	149 (16.5)	45 (13.8)	104 (17.9)	
T	1546 (90.4)	603 (92.8)	1043 (89.9)	0.02
C	164 (9.6)	47 (7.2)	117 (10.1)	
**rs2278426 (C/T)**				
CC	830 (91.7)	297 (91.4)	533 (91.9)	0.95
CT	72 (8.0)	27 (8.3)	45 (7.8)	
TT	3 (0.3)	1 (0.3)	2 (0.3)	
CT + TT	75 (8.3)	28 (8.6)	47 (8.1)	
C	1732 (95.7)	621 (95.5)	1111 (95.8)	0.45
T	78 (4.3)	29 (4.5)	49 (4.2)	

The two SNPs were in complete linkage equilibrium with linkage disequilibrium calculated as 0 (D was calculated as described in supplementary file SF1).

### Association of ANGPTL8 gene variants in the two study SNPs with anthropometric and metabolic parameters

To determine differences in the biochemical parameters based on ANGPTL8 gene variants, we analyzed these parameters in genotypes of rs737337 (T/C) and rs2278426(C/T) and the results are presented in Table 3. After controlling for age and sex, participants with TT genotype of rs737337 SNP showed significantly higher total cholesterol (p < 0.01) and LDL-cholesterol levels (p < 0.01) than those with TC and CC genotypes. Similarly, those with CC genotype of rs2278426 SNP showed significantly higher fasting glucose levels (p = 0.029), total-cholesterol levels (p = 0.004) and LDL-cholesterol levels (p < 0.001) than those with CT and TT genotypes.

**Table 3:** Anthropometric and biochemical parameters according to the two study SNPs.

rs737337 (T/C)						
Parameters	TT	TC	CC	p	TC + CC	p
N (M/F)	756 (364/392)	134 (69/65)	15 (8/7)		149 (77/72)	
Age (Years)	41.6 ± 10.6	39.9 ± 10.4	40.0 ± 7.1	0.24	39.9 ± 10.1	0.09
BMI (kg/m^2^)	29.9 ± 6.7	29.4 ± 7.2	27.7 ± 6.4	0.40	29.2 ± 7.1	0.32
Waist circumference (cm)	96.1 ± 18.6	95.7 ± 20.7	90.5 ± 15.1	0.54	95.7 ± 20.2	0.58
Hip circumference (cm)	105.9 ± 18.3	104.2 ± 19.8	100.9 ± 14.6	0.42	103.8 ± 19.3	0.24
WHR	0.90 ± 0.12	0.90 ± 0.15	0.90 ± 0.10	0.85	0.90 ± 0.12	0.58
SBP (mmHg)	120.1 ± 14.2	121.4 ± 16.1	122.9 ± 12.7	0.48	121.6 ± 15.7	0.25
DBP (mmHg)	77.1 ± 9.3	77.9 ± 10.2	78.2 ± 7.2	0.56	78.0 ± 9.9	0.29
Glucose (mmol/L)	5.6 ± 0.7	5.5 ± 0.8	5.2 ± 0.7	0.09	5.4 ± 0.8	0.07
Triglycerides (mmol/L) #	1.4 (1.0–2.0)	1.4 (1.0–2.1)	1.5 (1–1.7)	0.95	1.4 (1.0–2.1)	0.61
Total Cholesterol (mmol/L)	5.3 ± 1.1	**4.8 ± 1.1**^**a**^	**4.5 ± 1.0**^**a**^	**<0.01**	4.7 ± 1.1	**<0.01**
HDL-Cholesterol (mmol/L)	0.9 ± 0.4	0.9 ± 0.4	0.8 ± 0.3	0.61	0.9 ± 0.4	0.96
LDL-Cholesterol (mmol/L)	3.6 ± 0.9	**3.1 ± 1.0**^**a**^	**2.9 ± 0.9**^**a**^	**<0.01**	3.1 ± 1.0	**<0.01**
**rs2278426 (C/T)**						
**Parameters**	**CC**	**CT**	**TT**	**p**	**CT + TT**	**p**
N (M/F)	830 (402/428)	72 (37/35)	3 (2/1)		75 (39/36)	
Age (Years)	41.3 ± 10.7	41.2 ± 8.8	41.3 ± 7.2	0.99	41.2 ± 8.7	0.91
BMI (kg/m^2^)	29.8 ± 6.8	29.7 ± 6.8	29.4 ± 6.5	0.99	29.6 ± 6.8	0.95
Waist circumference (cm)	95.8 ± 18.7	97.6 ± 20.6	85.0 ± 19.5	0.48	96.9 ± 20.6	0.65
Hip circumference (cm)	105.7 ± 18.4	105.4 ± 19.4	99.7 ± 8.1	0.85	105.1 ± 19.0	0.83
WHR	0.90 ± 0.12	0.90 ± 0.16	0.85 ± 0.17	0.75	0.90 ± 0.16	0.95
Systolic BP (mmHg)	119.9 ± 14.4	123.7 ± 14.4	116.7 ± 23.1	0.10	123.5 ± 14.7	**0.05**
Diastolic BP (mmHg)	77.1 ± 9.4	79.6 ± 9.3	76.7 ± 11.5	0.10	79.5 ± 9.4	**0.04**
Glucose (mmol/L)	5.6 ± 0.8	**5.3 ± 0.8**^**b**^	5.4 ± 0.6	**0.03**	5.3 ± 0.7	**<0.01**
Triglycerides (mmol/L) #	1.4 (1.0–2.0)	1.5 (1.0–2.2)	1.7 (0.8–1.8)	0.67	1.5 (1.0–2)	0.57
Total Cholesterol (mmol/L)	5.2 ± 1.1	**4.8 ± 1.2**^**b**^	4.1 ± 0.9	**<0.01**	4.8 ± 1.2	**<0.01**
HDL-Cholesterol (mmol/L)	0.9 ± 0.4	0.9 ± 0.4	0.8 ± 0.1	0.84	0.9 ± 0.3	0.71
LDL-Cholesterol (mmol/L)	3.6 ± 1.0	**3.1 ± 1.1**^**b**^	2.7 ± 0.8	**<0.01**	3.1 ± 1.1	**<0.01**

Note: Data represented by Mean ± SD and Median (1st Q- 3rd Q) for Gaussian and non-Gaussian (#) variables respectively. P-Value denotes significance at <0.05. a & b represented significant difference compared to TT and CC in rs737337 and rs2278426 respectively.

### Association of ANGPTL8 gene variants with MetS components

Based on cut-off values, participants were divided as obese (BMI > 30), hypertensive (SBP/DBP > 130/85), low HDL (HDL-cholesterol <40 mg/dL for men and <50 mg/dL for women), hypertriglyceridemia (>1.7 mmol/l), hypercholesterolemia (>5.7 mmol/l), high glucose (>5.6 mmol/l) and MetS (IDF criteria) and analyzed the effect of ANGPTL8 gene variants on the above classified variables. A logistic regression analysis of the genotypes/alleles in the SNP rs737337 and SNP rs2278426 representing risk of having the different components of MetS are presented in Tables 4 and 5 respectively.

**Table 4:** Odds ratio (confidence interval 95%) representing MetS risk score with gene variants in rs737337 SNP.

Parameters	Yes	β	OR (95 % CI)^a^	p^a^	OR (95 % CI)^b^	p^b^
Obesity	465					
TT	397 (85.4)		1		1	
CC	6 (1.3)	−0.55	0.58 (0.20–1.64)	0.30	0.83 (0.26–2.61)	0.75
TC	62 (13.3)	−0.24	0.79 (0.54–1.15)	0.22	0.81 (0.55–1.19)	0.29
TC + CC	68 (14.6)	−0.27	0.77 (0.54–1.09)	0.14	0.81 (0.56–1.18)	0.27
T	856 (92.1)		1			
C	74 (7.9)	−0.28	0.76 (0.55–1.05)	0.10	0.83 (0.59–1.16)	0.27
Hypertension	296					
TT	244 (82.4)		1		1	
CC	5 (1.7)	0.06	1.06 (0.35–3.19)	0.92	1.75 (0.51–5.99)	0.37
TC	47 (15.9)	0.18	1.19 (0.80–1.77)	0.39	1.41 (0.92–2.17)	0.11
TC + CC	52 (17.6)	0.16	1.18 (0.81–1.72)	0.40	1.44(0.96–2.17)	0.08
T	535 (90.4)		1		1	
C	57 (9.6)	0.14	1.45(0.81–1.62)	0.44	1.42 (0.98–2.07)	0.066
Low HDL-C	589					
TT	490 (83.2)		1		1	
CC	11 (1.9)	0.37	1.44 (0.46–4.58)	0.53	1.02 (0.30–3.43)	0.97
TC	88 (14.9)	0.07	1.07 (0.72–1.59)	0.72	1.17 (0.78–1.78)	0.45
TC + CC	99 (16.8)	0.10	1.11 (0.76–1.61)	0.61	1.16 (0.78–1.72)	0.47
T	1068 (90.7)		1		1	
C	110 (9.3)	0.10	1.10 (0.78–1.55)	0.58	1.10 (0.77–1.57)	0.60
Hypertriglyceridemia	331					
TT	273 (82.5)		1		1	
CC	3 (0.9)	−0.82	0.44 (0.12–1.58)	0.21	0.57 (0.15–2.13)	0.40
TC	55 (16.6)	0.21	1.23 (0.85–1.79)	0.28	1.21 (0.83–1.78)	0.32
TC + CC	58 (17.5)	0.12	1.13 (0.79–1.62)	0.52	1.15 (0.79–1.66)	0.48
T	546 (89.5)				1	
C	64 (10.5)	0.03	1.03 (0.74–1.43)	0.87	1.07 (0.76–1.51)	0.70
Hypercholesterolemia	266					
TT	234 (88.0)		1		1	
CC	1 (0.4)	−1.84	0.16 (0.02–1.22)	0.08	0.20 (0.03–1.56)	0.12
TC	31 (11.7)	−0.40	0.67 (0.44–1.03)	0.07	0.70 (0.45–1.08)	0.11
TC + CC	32 (12.1)	−0.49	0.61 (0.40–0.93)	0.02	0.65 (0.42–0.99)	0.049
T	499 (93.8)		1		1	
C	33 (6.2)	−0.55	0.58 (0.39–0.86)	0.007	0.62 (0.41–0.93)	0.02
High Glucose	388					
TT	336 (86.6)		1		1	
CC	4 (1.0)	−0.86	0.42 (0.13–1.36)	0.15	0.58 (0.17–2.02)	0.40
TC	48 (12.4)	−0.36	0.70 (0.47–1.03)	0.07	0.68 (0.46–1.02)	0.06
TC + CC	52 (13.4)	−0.41	0.66 (0.45–0.97)	0.03	0.67 (0.46–0.99)	0.05
T	672 (93.3)		1		1	
C	56 (6.7)	−0.42	0.65 (0.46–0.93)	0.02	0.69 (0.48–0.98)	0.04
MetS	325					
TT	280 (86.2)		1		1	
CC	2 (0.6)	−1.34	0.26 (0.06–1.17)	0.08	0.33 (0.07–1.53)	0.16
TC	43 (13.2)	−0.22	0.80 (0.54–1.19)	0.27	0.84 (0.56–1.25)	0.39
TC + CC	45 (13.8)	−0.31	0.74 (0.50–1.08)	0.11	0.79 (0.53–1.16)	0.22
T	603 (92.8)		1		1	
C	47 (7.2)	−0.36	0.69 (0.49–0.99	0.04	0.75 (0.52–1.08)	0.12

Note**:** OR^a^ and p^a^ represents the univariate values while OR^b^ and p^b^ represents the values after adjustment with age and gender. Obesity was considered present if BMI > 30 kg/m^2^; hypertension if blood pressure ≥130/85 mmHg; low HDL-C if HDL-cholesterol <40 mg/dL for men and <50 mg/dL for women; hypertriglyceridemia if triglycerides levels ≥1.7 mmol/L; hypercholesterolemia if total cholesterol>5.7 mmol/l and high glucose if fasting glucose levels ≥5.6 mmol/L. MetS was considered present based on the IDF criteria. P < 0.05 was considered significant.

**Table 5:** Odds ratio (confidence interval 95%) representing MetS risk score with gene variants in rs2278426 SNP.

Parameters	Yes	β	OR (95% CI)^a^	p^a^	OR (95% CI)^b^	p^b^
Obesity	465					
CC	428 (92.0)		1		1	
TT	2 (0.4)	0.58	1.79 (0.16–13.9)	0.63	1.66 (0.15–15.5)	0.68
CT	35 (7.5)	−0.14	0.87 (0.54–1.42)	0.58	0.94 (0.56–1.55)	0.80
CT + TT	37 (7.9)	−0.11	0.90 (0.56–1.44)	0.66	0.96 (0.58–1.58)	0.87
C	891 (95.8)		1		1	
T	39 (4.2)	−0.08	0.93 (0.59–1.46)	0.74	0.98 (0.61–1.58)	0.94
Hypertension	296					
CC	263 (88.9)		1		1	
TT	2 (0.7)	1.36	3.89 (0.35–23.1)	0.27	3.94 (0.34–28.78)	0.27
CT	31 (10.5)	0.54	1.72 (1.04–2.86)	0.035	2.03 (1.17–3.51)	0.01
CT + TT	33 (11.2)	0.58	1.79 (1.10–2.93)	0.02	2.09 (1.23–3.58)	0.007
C	557 (94.1)		1		1	
T	35 (5.9)	0.59	1.80 (1.12–2.89)	0.015	2.09 (1.25–3.48)	0.005
Low HDL-C	589					
CC	541 (91.9)		1		1	
TT	3 (0.5)		—		—	
CT	45 (7.6)	−0.12	0.89 (0.54–1.45)	0.65	0.85 (0.51–1.43)	0.56
CT + TT	48 (8.1)	−0.05	0.95 (0.58–1.55)	0.84	0.92 (0.55–1.53)	0.74
C	1127 (95.7)		1		1	
T	51 (4.3)	0.01	1.01 (0.63–1.63)	0.95	0.99 (0.60–1.62)	0.95
Hypertriglyceridemia	331					
CC	300 (90.6)		1		1	
TT	1 (0.3)	−0.12	0.88 (0.08–9.7)	0.92	0.84 (0.08–9.3)	0.89
CT	30 (9.1)	0.23	1.26 (0.77–2.06)	0.35	1.28 (0.77–2.13)	0.34
CT + TT	31 (9.4)	0.22	1.25 (0.77–2.01)	0.37	1.26 (0.77–2.10)	0.36
C	630 (95.2)		1		1	
T	32 (4.8)	0.20	1.22 (0.77–1.93)	405	1.23 (0.76–1.98)	0.40
High Glucose	388					
CC	366 (94.3)		1		1	
TT	1 (0.3)	−0.62	0.54 (0.05–5.93)	0.61	0.50 (0.05–5.60)	0.58
CT	21 (5.4)	−0.67	0.51 (0.30–0.88)	0.015	0.52 (0.29–0.88)	0.016
CT + TT	22 (5.7)	−0.67	0.51 (0.30–0.87)	0.01	0.51 (0.29–0.87)	0.01
C	753 (97.0)		1		1	
T	23 (3.0)	−0.64	0.53 (0.32–.87)	0.01	0.52 (0.31–.87)	0.015
Hypercholesterolemia	266					
CC	247 (92.9)		1			
TT	0 (0.0)	—	—			
CT	19 (7.1)	−0.17	0.85 (0.49–1.46)	0.55	0.88 (0.51–1.54)	0.66
CT + TT	19 (7.1)	−0.22	0.80 (0.47–1.38)	0.42	0.83 (0.48–1.44)	0.51
C	513 (96.4)		1		1	
T	19 (3.6)	−0.27	0.77 (0.45–1.29)	0.32	0.79 (0.46–1.35)	0.38
MetS	325					
CC	297 (91.4)		1		1	
TT	1 (0.3)	−0.11	0.90 (0.08–9.90)	0.93	0.85 (0.08–9.43)	0.89
CT	27 (8.3)	0.07	1.08 (0.66–1.77)	0.77	1.15 (0.69–1.91)	0.60
CT + TT	28 (8.6)	0.07	1.07 (0.66–1.74)	0.79	1.13 (0.68–1.87)	0.63
C	621 (95.5)		1		1	
T	29 (4.5)	0.06	1.06 (0.66–1.69)	0.81	1.11 (0.69–1.80)	0.66

Note**:** OR^a^ and p^a^ represents the univariate values while OR^b^ and p^b^ represents the values after adjustment with age and gender. Obesity was considered present if BMI > 30 kg/m^2^; hypertension if blood pressure ≥130/85 mmHg; low HDL-C if HDL-cholesterol <40 mg/dL for men and <50 mg/dL for women; hypertriglyceridemia if triglycerides levels ≥1.7 mmol/L; hypercholesterolemia if total cholesterol>5.7 mmol/l and high glucose if fasting glucose levels ≥5.6 mmol/L. MetS was considered present based on the IDF criteria. P < 0.05 was considered significant.

The TC + CC genotype and C allele of rs737337 were associated with decreased risk of hypercholesterolemia in the univariate model as well as after adjustment with age and gender [Odd ratio (OR) 0.61, 95%CI: 0.40–0.93, p = 0.02 and OR 0.58, 95% CI: 0.39–0.86, p = 0.007 respectively for univariate; and OR 0.65 (0.42–0.99), p < 0.05 and OR 0.62 (0.41–0.93), p = 0.02 respectively after adjustment]. In addition, they were also associated with decreased risk of hyperglycemia in both models (OR 0.66, 95% CI: 0.45–0.97, p = 0.03 and OR 0.65, 95% CI: 0.46–0.93, p = 0.02 respectively for TC + CC and C alleles in univariate model; and OR 0.67 (0.46–0.99), p < 0.05 and 0.69 (0.48–0.98), p = 0.04 respectively in the adjusted model). The c-allele of rs737337 was also found to be associated with decreased risk of full MetS in the univariate model, however lost statistical significance in the adjusted model (OR 0.69, 95% CI: 0.49–0.99,p = 0.04 in the univariate model and OR 0.75 (0.52–1.08,p = 0.12 in the adjusted model). (Table 4).

Similarly, CT, CT + TT genotype and T allele of rs2278426 is associated with increased risk of hypertension in both models (OR 1.72 (1.04–2.86), p = 0.04; OR 1.79 (1.10–2.93), p = 0.02; and OR 1.80 (1.12–2.89), p = 0.02 respectively in the univariate model; and OR 2.03 (1.17–3.51), p = 0.01; OR 2.09 (1.23–3.58), p < 0.01; and OR 2.09 (1.25–3.48), p < 0.01 respectively in the adjusted model. The same genotypes and allele of rs2278426 were, however, associated with decreased risk of elevated serum glucose levels in both the models (p < 0.05) (Table 5).

**Figure 1:**
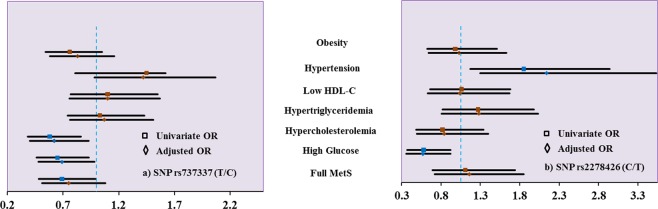
Association of SNPs rs rs737337 (C versus T, 1a) and rs2278426 SNP (T versus C, 1b) with individual components of MetS. Note: The data is represented by the odds ratio and its 95% confidence interval. The data was adjusted for age and gender in the adjusted model.

Figure 1 summarizes the results of the association of SNP rs737337 (1a) and rs2278426 with individual components of MetS.

## Discussion

The low-grade inflammatory state of MetS and its components like dyslipidemia, hyperglycemia, hypertension and obesity has a more severe outcome of 5-fold and 2-fold increase respectively in T2DM and cardiovascular diseases^19,20^. To address the public issue posed by MetS and associated complications, researchers in the recent past have been focusing on the preventive and therapeutic strategies to combat this menace. ANGPTL8 seems to be one such strategy and recent studies have demonstrated that the circulating levels of ANGPTL8 may be important in the course of development of MetS and its components^21^. Research studies are limited to the association of the circulating levels of ANGPTL8 and MetS state, and the investigations on the associations of ANGPTL8 gene variants with MetS and its individual components are limited particularly in Saudi Arabia.

In this study, we examined whether the polymorphism of ANGPTL8 SNPs rs2278426 (C/T) and rs737337 (T/C) play a role in the increased risk of MetS in Saudi Arab adults. As rs2278426 and rs737337 variants have been found to be associated with HDL-C levels and lipoprotein metabolism, which provide novel support for functional consequences of the both variants. To establish the link between MetS and SNPs of ANGPTL8 rs2278426 and rs737337, we identified the frequency and distribution of SNPs in the studied participants and evaluated their association with MetS phenotypes. Our findings demonstrated that variant C allele in rs737337 is associated with decreased risk of hypercholesterolemia and hyperglycemia; and the variant T allele in rs2278426 is associated with decreased risk of hyperglycemia and these associations remained statistically significant even after adjustment with age and sex.. Interestingly, our study also pointed out that allele of rs2278426 was associated with increased risk of hypertension even after adjustment.

ANGPTL8 or betatrophin is a novel liver and adipose tissue secreted protein that is nutritionally regulated. In humans, betatrophin is liver specific and its expression has been found to be relatively higher than in other tissues, while in mouse it is enhanced in both liver and adipose tissue^16^. ANGPTL8 is known to play a key role in lipid metabolism^22^, and our study showed that ANGPTL8 gene variants were significantly associated with total cholesterol and TG levels. Also, the odds for having hypertension significantly increased for the T allele of SNPs rs2278426 (C/T). This is in line with previous studies^23^ which showed ANGPTL8 levels higher in hypertensive subjects compared to non-hypertensive subjects. This association with hypertension may be explained by a strong correlation of ANGPTL8 with high sensitive C-reactive protein (HsCRP) found in some studies^24^. HsCRP with oxidized LDL have been shown to form complexes that promote atherosclerosis in diabetic mice leading to vascular inflammation and development of hypertension^25,26^. The association between betatrophin and hypertension indicates that its plasma level might be involved in the risk of cardiovascular events and could be regarded as a predictive biomarker for early diagnosis and management. It will be of interest though to validate and strengthen our findings, by carrying out the same study in a larger size population and or in two different Saudi populations to deepen our understanding of the association of ANGPTL8 SNPs rs2278426 and rs737337 with MetS.

Homology of ANGPTL8 with ANGPTL3’s and ANGPTL4’s N-terminal domains are necessary for lipid regulation through LPL binding^27^. Moreover, our data showed that lipid levels were significantly higher in MetS subjects. Zhang et al. found that overexpression of betatrophin has been shown to increase serum triglycerides, whereas recombinant betatrophin inhibits LPL activity^16^. Furthermore, LPL inhibition in cardiac and skeletal muscle have been linked with ANGPTL8^28^. In addition, some studies reported that ANGPTL8 co-expression with ANGPTL3 further increases serum TG showing a positive association^29,30^. Among various ethnic groups, significant differences in the allele frequencies of ANGPTL8 gene variants and their association with concentration of blood lipids have been reported. In Hispanic and African Americans, for example, the T allele of rs2278426 was reported to be associated with lower levels of LDL-C, while in European Americans this association was absent^14^. Similarly, a study showed a significant inverse correlation between ANGPTL8 and TG in patients with T2DM^31^, while other reports showed no association^32^. Thus, while it is clear that deregulated lipid metabolism through ANGPTL8 may be in part responsible for the observed association of ANGPTL8 gene variants with MetS, more studies are needed to uncover the pathophysiological pathways that involves betatrophin in the in the development of MetS.

In addition to its role in lipoprotein metabolism, ANGPTL8 is a potential target for β-cell regenerative therapy in diabetes due to its ability to induce β-cell proliferation^33^, suggesting that decreases in ANGPTL8 levels or function may worsen glucose tolerance. Previous studies demonstrated that betatrophin was up-regulated in T2DM individuals, which was assumed to due to increase in insulin production through increasing β-cell proliferation^34,35^. Although earlier reports stressed that ANGPTL8 expression gets induced by insulin, however, there are reports that suggest no association between ANGPTL8 and β-cell proliferation as mice lacking ANGPTL8 showed normal glucose metabolism under insulin resistance conditions^9^. In agreement with the associations found in this study, Abu-Farha et al. in his study while examining the changes in circulating ANGPTL8 levels in a large cohort of 1049 non-diabetic people and 556 people with T2DM found positive association with fasting glucose and DM duration^36^. In this study, the C-allele and T-allele of SNPs rs737337 (T/C) and rs rs2278426 (C/T) are associated with decreased risk of hyperglycemia. The exact mechanism by which sequence variation exerts its effect has not been established, it may be possible that the ANGPTL8 protein structure could be affected due to the variation in the charge of the amino acid residue from arginine to tryptophan. Research findings still suggest a role for ANGPTL8 in glucose metabolism apart from β-cell proliferation, however, to confirm that it will require further research into the physiological factors that regulate ANGPTL8 expression.

In the present study, older participants exhibited the typical characteristics of MetS including central obesity. They also showed high blood pressure, elevated glucose and triglyceride levels and decreased HDL-cholesterol levels. Also, the variant C-allele of SNP rs737337 (T/C) showed decreased risk of the full MetS. This is in agreement with studies showing increased expression of betatrophin in T2DM and/or obese subjects^32,34^. Furthermore, there is accumulating evidence that showed positive associations between circulating betatrophin levels, insulin resistance and T2DM^37,38^. The discrepancy in the study^39^ that showed that ANGPTL8/betatrophin concentrations was decreased in human obese subjects as well as subjects with T2DM was probably because of the kit used to measure betatrophin. Other studies reported non-significant differences in betatrophin levels in subjects with or without DM^13,40^. Fu et al. suggested that the reason for these discrepancies may be because of different sample size, BMI, age and ethnic group; and different ELISA kits employed^34^, however, further investigations are required to elucidate these discrepancies.

Despite these interesting findings reported in this study, we acknowledge certain limitations. First of all the investigation was done on a specific population comprising of Saudi Arabian adults and the findings should be used with caution when extrapolated to other ethnicities. Secondly, the study focused on the gene variants in the two SNPs rs737337 and rs 2278426 with the risk of full MetS and its individual components and lacked the data on the circulating levels of betatrophin and insulin in the studied subjects. The association of circulating levels of betatrophin and/or insulin resistance with MetS, thus, cannot be concluded in Saudi adults from this study and probably needs a separate investigation. Also, because of the difficulty in assuming the prevalence of the minor alleles of a SNP in the two study groups particularly when there is a limited literature available for the concerned population, it is hard to estimate a sufficient sample size for a genotype association study. Keeping this in mind, the authors recommend to validate the results observed here in a bigger sample size and if possible in two different populations. Nonetheless, with its limitations as mentioned, the findings in this study are still of interest not only in terms of the association of gene variants of a novel glycoprotein, ANGPTL8 with the disorders (MetS and its components) that has global health outcome but also as a stepping stone for future such studies.

## Conclusion

In conclusion, the study investigated the gene variants in SNPs rs 737337 (T/C) and rs 2278426 (C/T) in association with the risk of MetS and its individual components in Saudi adults and found that these variants are associated with lower risk of hypercholesterolemia and hyperglycemia as components of MetS. These findings supplement the growing literature supporting the role of ANGPTL8 in lipid and glucose metabolism, the disorder of which contributes to the development of MetS, however at the same time recommends that the pathophysiological upregulation of betatrophin in people with MetS needs further investigation to underline the corresponding molecular mechanisms.

## Research Design and Methods

### Subjects

Sample size estimation for this genetic association study was done by using G*power 3.1.9.4 software. For a two-tailed Fischer exact test in two independent groups with α = 0.05; power 0.80; proportion 1 as 0.35 and allocation ratio of 0.54 (since prevalence of metabolic syndrome in Saudi Arabia is 35% as per the literature); and assuming a 10% difference of risk alleles in cases and controls we expected another proportion as 0.45. When these values were entered, a sample size of 847 was calculated. Based on this, a total of nine hundred and five unrelated Saudi adults [580 healthy controls (296 males and 284 females) and 325 MetS (145 males and 185 females)] were randomly selected from the database of Biomarker Screening conducted by Chair for Biomarker of Chronic Diseases (CBCD) in King Saud University (KSU), Riyadh, Saudi Arabia, a capital-wide epidemiologic study for Saudis^41^. In brief, using a cluster sampling strategy, participants were recruited from different regions in and around the Riyadh region. An overnight fasting blood sample was taken from the participants at the associated primary health care center (PHCC) where anthropometric measurements and data related to general demographic information; past and present medical history etc. was also recorded. Before including in this study, a written informed consent was collected from all participants. The study was approved by Ethics Committee at the College of Science, KSU, Riyadh, Saudi Arabia.

For this study, MetS screening was done based on the criteria by International Diabetes Federation (IDF) which included components like central obesity (waist circumference ≥102 cm/≥88 cm for men/women) plus 2 or more factors: hypertriglyceridemia (triglycerides ≥ 1.7 mmol/L); low HDL-cholesterol (<40/<50 mg/dL for men/women); hypertension (blood pressure ≥130/85 mmHg) and hyperglycemia (fasting glucose levels ≥5.6 mmol/L).

### Anthropometry, blood collection and biochemical analysis

Anthropometric data was taken from the database. This comprised of height (centimeters), weight (kg), waist and hip circumference (centimeters), and blood pressure (in mmHg). To calculate BMI, we used the formula weight in kg/(height in meter)^2^. Fasting whole blood and serum samples collected at the recruitment were transported at proper conditions to the biobank facility of CBCD, aliquoted there and kept at suitable storage conditions for future analysis. Glucose, total cholesterol, HDL-cholesterol and triglycerides as biochemical analysis were measured using routine commercially available kits on an automated biochemical analyzer (Konelab, Espoo, Finland). The procedure of biochemical analysis was already published in detail^42^. Whole blood samples were used for genetic analysis.

### Genetic analysis

Genomic Prep mini spin kit (GE healthcare, NJ, USA) was utilized to isolate DNA from the whole blood samples according to the protocol. A Nano-drop ND 1000 spectrophotometer was used to check the concentration and purity (260/280 nm) of extracted DNA. Pre-designed TaqMan genotyping assays (Applied Biosystems, CA, USA, rs737337 Assay ID: C___8727006_10; and rs2278426 Assay ID: C__15965472_10) were employed in a real time PCR to evaluate the allelic discrimination in the two tagging SNPs (rs737337 and rs2278426). The TaqMan assay IDs (primers and probes) are given in Table 6.

**Table 6:** TaqMan Assay IDs Used.

dbSNP	Assay ID	Context Sequence [VIC/FAM]	Assembly Location
rs737337	C_8727006_10	**CCTGGGGGTGCACAGAGGACACGGC[C/T]GTGAGCTCCACACTGAACACGCCCT**	ch. 19: 11347493
rs2278426	C_15965472_10	**CGGTGTGTACAGGACCACGGAGGGA[C/T]GGCTGACAAAGGCCAGGAACAGCCT**	ch. 19: 11350488

10 µL of volume containing 1X TaqMan genotyping Master Mix (Applied Biosystems), 1X mix of unlabeled PCR primers and TaqMan probes, and 30 ng of template DNA were mixed in 96-well PCR plates for amplification and a Bio-Rad CFX96 Real-Time PCR Detection System (Bio-Rad, Milan, Italy) was utilized for its detection. Thermal cycling utilized a denaturation step of 10 min at 95 °C, which was followed by 45 cycles of 15 s at 95 °C and 90 s at 60 °C. The software used for allelic discrimination analysis was from Bio-Rad (CFX Manager Software, Version 1.6). Dedicated PCR pipettes and reagents were only employed for all PCR reactions. A validation step of re-genotyping of 40 random samples was done and the results were found to be reproducible.

### Statistical analysis

SPSS (version 22.0, IBM, NY, USA) was used to analyze the data. Continuous Gaussian variables were shown as mean ± standard deviation (SD) and continuous non-Gaussian variables were shown as median (Q1-Q3). Genotype distributions between the MetS and control subjects were reported as allele frequency (%) and the difference was computed using Chi-square test of distributions. Odds ratios (ORs) and 95% confidence intervals (CIs) depicting the risk of metabolic syndrome and its individual components vis a vis various alleles in the two studied SNP’s were calculated using multinomial logistic regression with genotypes as the factor; sex and age as covariates; and most common genotypes as reference. P-value <0.05 was considered significant in all tests (two-tailed).

### Statement of Informed consent

Written informed consent was obtained from each participant in this study.

### Details of Ethics Approval

All the procedures followed in this study were in accordance with the ethical standards of the responsible committee on human experimentation (institutional and national) and with the Helsinki Declaration of 1975, as revised in 2008. The study was approved by the Ethics Committee of College of Science, King Saud University.

## Supplementary information

Supplementary Information.

## Data Availability

The dataset analysed for getting the results observed in this study is available from the corresponding author on reasonable request.
