# Supplementary material for: Association of ANGPTL8 (Betatrophin) Gene Variants with Components of Metabolic Syndrome in Arab Adults
Source: Sci Rep. 2020 Apr 21;10:6764. doi: 10.1038/s41598-020-63850-7 (PMC7174409; doi:10.1038/s41598-020-63850-7)
Supplement: Supplementary file 1 — Supplementary Information. [file 41598_2020_63850_MOESM1_ESM.pdf]

Association of ANGPTL8 (Betatrophin) Gene Variants with Components of Metabolic Syndrome in Arab Adults

Amal Alenad[1,2], Mona M. Al-Onazi[1], Majed S. Alokail[1,2], Kaiser Wani[2], Abdul Khader Mohammed[3], Abdullah M. Alnaami[2], Maha Sulimani[1], Seema Zargar[1], Mario Clerici[2,4,5], Nasser M. Al-Daghri[1,2*]

Supplementary File 1. Linkage Disequilibrium between SNPs rs737337 and rs2278246

Allele frequency for A (rs737337) and B (rs2278246)

Allele		Frequency
A(T)	p1	0.9041
A-C	p2	0.0959
B(T)	q1	0.0431
B(C)	q2	0.9569

Haplotype frequency for A(rs737337) and B (rs2278246)

**Haplotype**		**Frequency**
A(T)B(T)	x11	0.0390
A(T)B(C)	X12	0.8651
A(C)B(T)	X21	0.0041
A(C)B(C)	X22	0.0918

Calculation of linkage disequilibrium (D)

D = (x11)*(x22) minus (x12)*(x21)
D= (0.390)*(0.0918) minus (0.8651)*(0.0041)
D=0
$R^2 = D^2/p1*p2*q1*q2 = 0$
